# Supplementary material for: Curcumin Reprograms TAMs from a Protumor Phenotype towards an Antitumor Phenotype via Inhibiting MAO-A/STAT6 Pathway
Source: Cells. 2022 Nov 2;11(21):3473. doi: 10.3390/cells11213473 (PMC9655729; doi:10.3390/cells11213473)
Supplement: Supplementary file 1 [file cells-11-03473-s001.zip › cells-1930867-supplementary.pdf]

Figure 3C

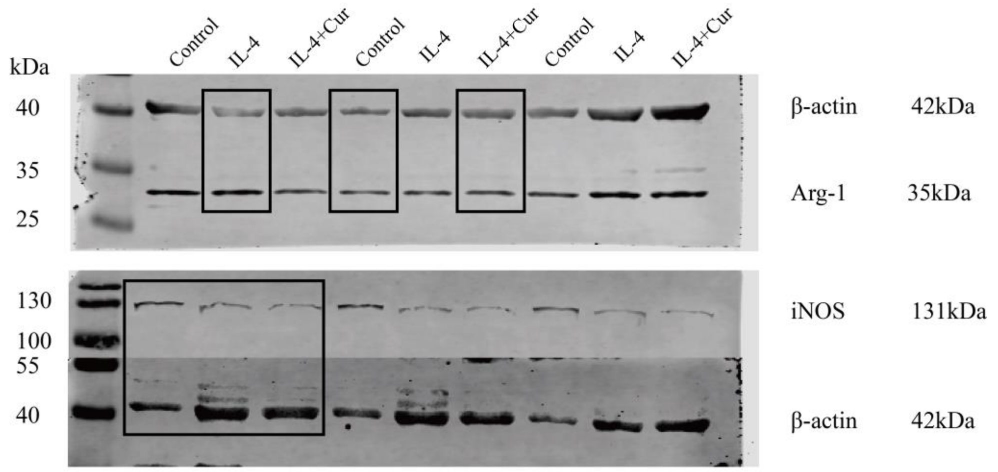

Figure 5B

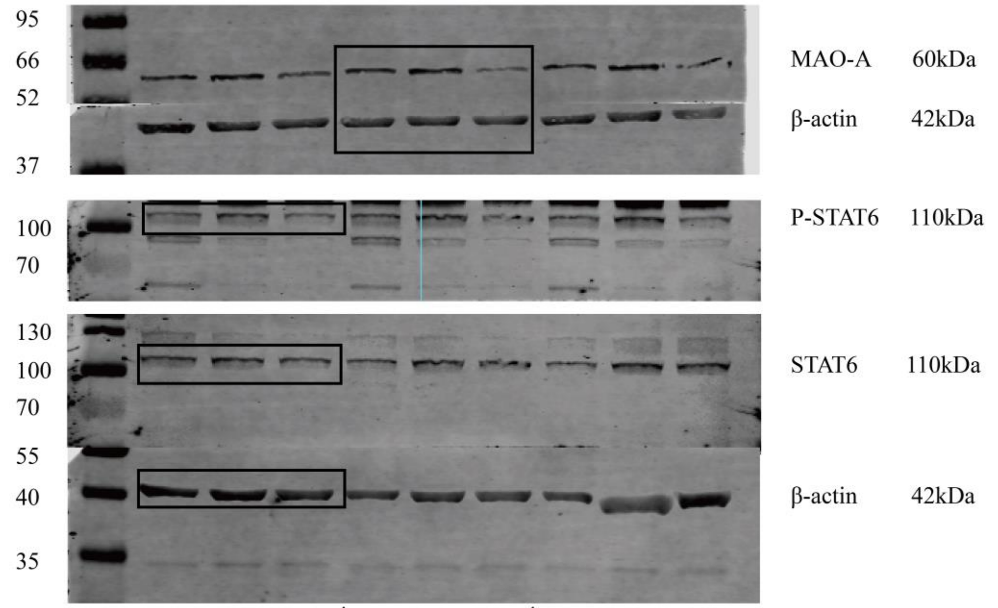

Figure 4H

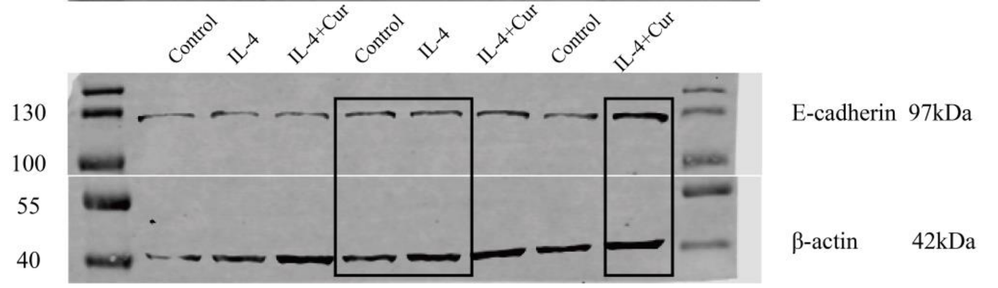

Figure S1. Original images of Western blot. The images which are used in the manuscript are marked with boxes.

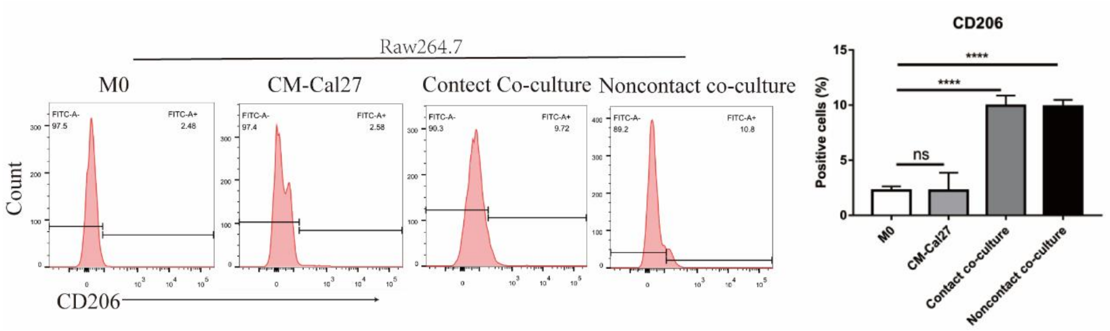

Figure S2. Flow cytometric analysis of surface markers of CD206 in Raw264.7 cells polarized by various methods. Data are represented as mean±SD (n=3). P values were determined by one-way analysis of variance (ANOVA). *P < 0.05; **P < 0.01; ***P < 0.001.

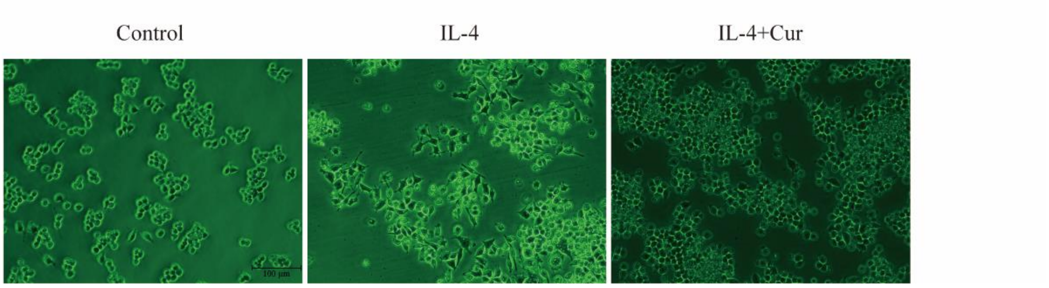

Figure S3. Representative micrographs of morphology changes of Macrophages.

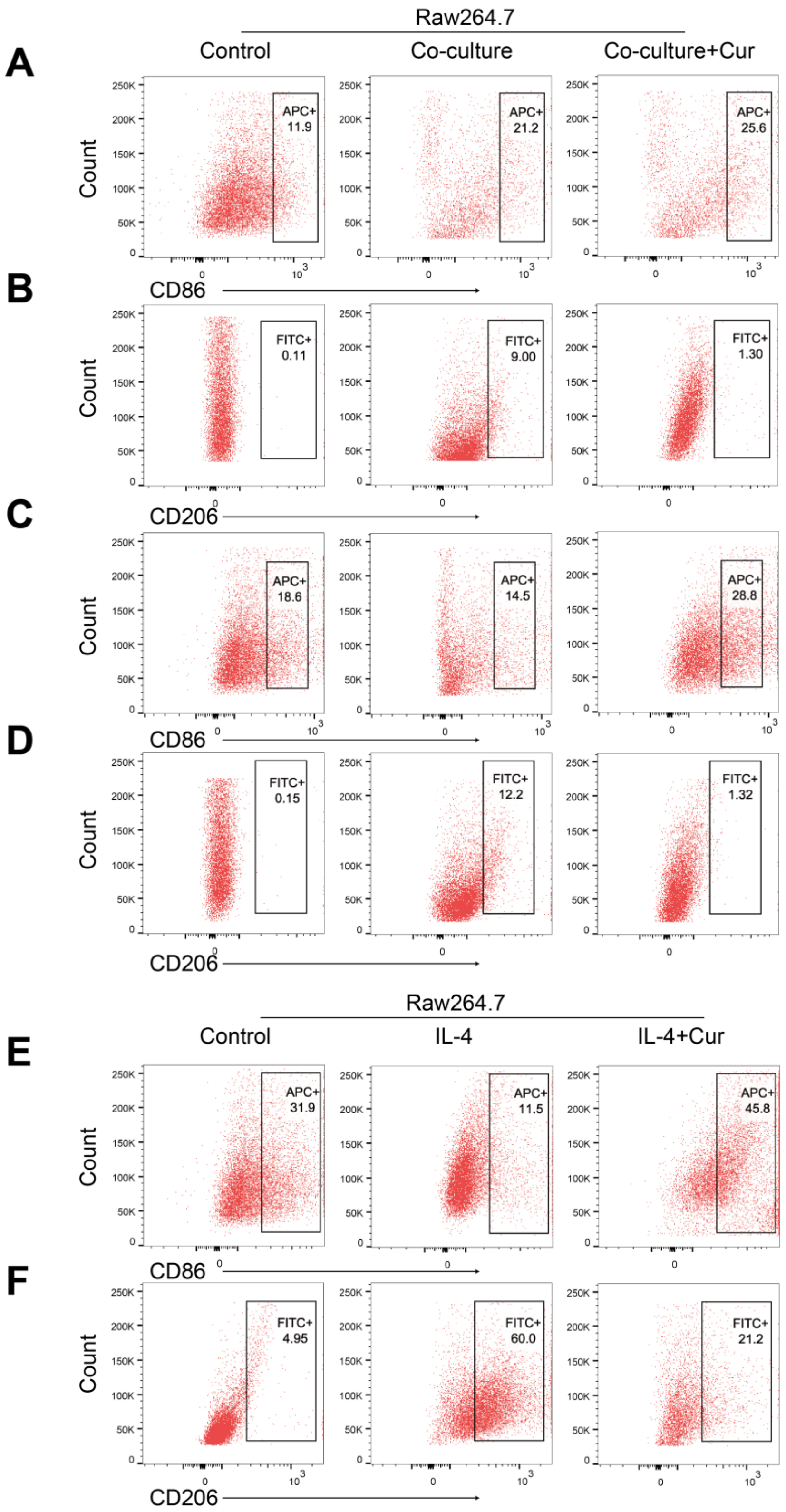

Figure S4. FACS results in the above figure A–B, C–D, and E–F also have been presented in the form of histograms in the revised manuscript (Figure 2B, D), (Figure 2F, H), and (Figure 3G, I).
